# Anthocyanin-Loaded Polymers as Promising Nature-Based, Responsive, and Bioactive Materials

**DOI:** 10.3390/polym16010163

**Published:** 2024-01-04

**Authors:** S.S. Rosales-Murillo, Julia Sánchez-Bodón, S.L. Hernández Olmos, M.F. Ibarra-Vázquez, L.G. Guerrero-Ramírez, L. Pérez-Álvarez, J.L. Vilas-Vilela

**Affiliations:** 1 Chemistry Department, University Center of Exact Sciences and Engineering, University of Guadalajara, Guadalajara 44430, Mexico; sil.soledad024@gmail.com (S.S.R.-M.); saira.hernandez@academicos.udg.mx (S.L.H.O.); maria.ibarra4054@academicos.udg.mx (M.F.I.-V.); lguillermo.guerrero@academicos.udg.mx (L.G.G.-R.); 2 Macromolecular Chemistry Group (LQM), Physical Chemistry Department, Faculty of Science and Technology, University of the Basque Country, 48940 Leioa, Spain; julia.sanchez@ehu.eus (J.S.-B.); joseluis.vilas@ehu.eus (J.L.V.-V.); 3 Technological University of Jalisco, Guadalajara 44970, Mexico; 4 BCMaterials, Basque Center for Materials, Applications and Nanostructures, UPV/EHU Science Park, 48940 Leioa, Spain

**Keywords:** anthocyanins, polymers, food packaging, healthcare

## Abstract

Anthocyanins are a specific group of molecules found in nature that have recently received increasing attention due to their interesting biological and colorimetric properties that have been successfully applied in several fields such as food preservation and biomedicine. Consequently, reviews devoted to a general overview of these flavonoids have proliferated in recent years. Meanwhile, the incorporation of anthocyanins into polymeric systems has become an interesting strategy to widen the applicability of these molecules and develop new smart and functional polymers in the above cited areas. However, anthocyanin-based polymers have been scarcely reviewed in the literature. Accordingly, this review aims to be a systematic summary of the most recent approaches for the incorporation of anthocyanins into macro-, micro-, or nanostructured polymers. Moreover, this work describes the fundamentals of the applicability of smart anthocyanin-based polymers and offers an updated review of their most interesting applications as sensors, biological regulators, and active materials.

## 1. Introduction

Anthocyanins are non-toxic flavonoids pigments that are widely distributed in nature, offering attractive colors to the flower petals and fruits of some plants, such as bright orange, pink, scarlet, red, mauve, violet and blue. The term “anthocyanin” comes from the Greek words anthos (“flower”) and kyanos (“blue”), which was originally used to describe the blue pigment in cornflowers. Anthocyanins are positively charged plant-derived molecules classified as the largest group of water-soluble pigments. They can be found accumulated in the vacuoles of epidermal or subepidermal cells in plant organs such as roots and leaves [1]. They are present in fruits such as blueberries, strawberries, bananas, apples, blackberries, and grapes, to name a few, as well as in vegetables like purple cabbage, red onions, and eggplants [2].

These compounds belong to the flavonoid family. Flavonoids are crucial for plant development and proper functioning due to their role in attracting animals for oviposition and protecting against UV light or infections by phytopathogenic organisms [3]. In addition, flavonoids present remarkable properties related to human health, including antioxidant, anti-inflammatory, antidiabetic, chemopreventive, and antimutagenic properties, among others [4]. Consequently, their ongoing consumption is regarded as a beneficial health promotion practice and a preventive measure against numerous diseases. However, anthocyanins face a challenge in terms of absorption by the human body. The body rapidly metabolizes and excretes anthocyanins, reducing their beneficial activity [5]. Beyond their low bioavailability, anthocyanins also have low stability and are highly sensitive to external factors, making them susceptible to decomposition [6].

**Figure 1:**
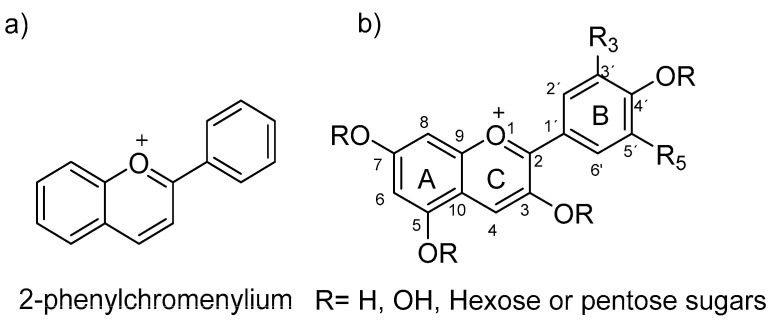
(**a**) Flavylium ion structure; (**b**) common numbering of flavylium ion.

Within anthocyanins’ characteristics, they are known to be water- and alcohol-soluble organic compounds that have a three-ring heteroaromatic polyphenolic skeleton. Anthocyanins are glycosylated analogues, mainly at the C3 position, of anthocyanidins, both based on the 2-phenyl-benzopyrilium chromophore structure (flavylium ion in Figure 1), which shows an extended π conjugation, as well as the presence of a positive charge and several free -OH groups. These characteristics allow anthocyanins to absorb light in the visible region, which generates a great variety of dye colors, making them one of the most important natural pigments besides chlorophyll.

Anthocyanidins possess an almost planar chemical structure characterized by variations in the number and positioning of hydroxyl groups and/or methoxy groups. These structural differences play a crucial role in determining the natural availability of a diverse array of anthocyanins and anthocyanidins [7]. Anthocyanidin glycosides are 3-monoglucosides and 3,5-diglucosides, consisting of the most common sugar, glucose; however, rhamnose, xylose, galactose arabinose, and rutinose can also appear [8].

**Figure 2:**
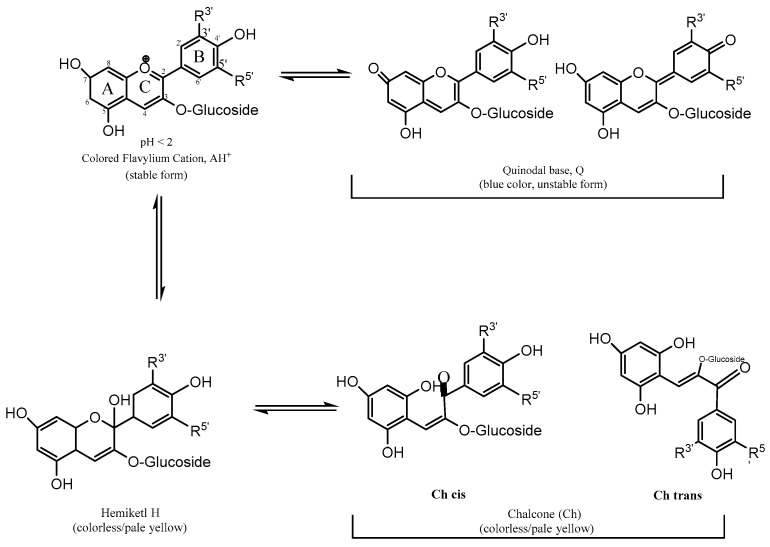
pH-dependent structural changes and color variation of anthocyanins in aqueous solution. Reprinted with permission from Calogero et al. [8]. Copyright (Calogero G) (Chem Soc Rev).

Depending on conditions such as temperature, light, solvent, metal ions, and mainly pH, anthocyanins undergo structural modifications accompanied by photophysical and chemical changes. In fact, different proton concentrations can lead these molecules to hydrated open ring or quinoidal forms [9]. Anthocyanins are very stable at acidic pHs, but as the pH approaches neutral, their stability dramatically decreases, leading to complete degradation at pHs above 7. The kinetics of these changes is one of the main factors that determines the final color of these compounds (Figure 2) [10].

Generally, a molecule with more -OH groups generates a more intense blue color, while in those with -OR groups, the coloration turns red. As for the glycosyl substituents, their presence decreases the coplanarity of the B-ring, so they tend to be less stable, and the coloration is usually not as intense.

If, in addition to sugar, there is an acyl radical in the molecule, they are called acylated anthocyanins. An increase in glucosidic substitution and acylation with cinnamic acids allows them to be more stable and retain their characteristic color at alkaline pHs [11].

Anthocyanins have received increasing attention from researchers in recent years mainly due to their importance in two main application sectors: the food industry and healthcare. Regarding food technology, the utility of anthocyanins extends beyond their role as a natural colorant to enhance the organoleptic properties of food. Their inherently reactive nature has led to their consideration as colorimetric indicators for assessing food quality in advanced smart food packaging technologies. Regarding healthcare and pharmaceutical applications, a large amount of highly interesting health-promoting effects of anthocyanins have been revealed in the last decade. Thus, the nutraceutical effect of the anthocyanin or anthocyanin-rich food intake has attracted research interest. However, their low stability and bioavailability hinder an efficient effect of anthocyanins in the human body. Consequently, research efforts have focused on the development of strategies to improve their stability, bioavailability, and color preservation. Among these strategies, encapsulation has become the most effective and explored one. Encapsulation is based on the development of a protective coverage of anthocyanins, which, in addition to improving their stability and bioavailability, could also provide advanced properties or performance (like selective or controlled release).

Accordingly, there exists a real need for immobilizing anthocyanins within appropriate substrates that fulfill the wide range of physical and chemical requirements specified for the mentioned applications. Among these substrate systems, polymers are versatile materials that show unique properties in a wide range of forms, like films, emulsions, hydrogels, mats, and nanoparticles, among others, that are of great interest in the food-packaging and healthcare sectors. In the light of this combination, the incorporation of anthocyanins within polymeric matrices has proliferated in recent years as a promising approach to utilize the synergistically positive properties of both substrates and active agents.

Until now, several works have reviewed anthocyanin food applications, the physiological effects of anthocyanins as functional food ingredients, as well as anthocyanins applications in health and disease prevention. However, despite there being a considerable number of studies reporting anthocyanin–polymer combinations that warrant highlighting, this specific field lacks a systematic and summarizing review. The objective of this paper is to summarize the most recent advances in the investigation of anthocyanins incorporated into polymers, emphasizing those related to food and healthcare applications. This combination of anthocyanin and polymer matrices is not only a promising alternative to solve the real challenge of the encapsulation of these phytochemicals, but it allows the development of smart biosensors and active materials, which highlights the relevance of the scope of this review.

## 2. Extraction Procedures for Anthocyanins

Due to their compositional complexity, the extraction from biological matrices and the selective isolation of a specific anthocyanin with chemical features is not a trivial issue. Consequently, it has become the main research target in this field. Moreover, several reviews exist on the methods used to extract and purify anthocyanins [1].

The conventional method for extracting anthocyanins is through maceration according to their polar nature. The use of polar solvents like methanol and ethanol makes the extraction of anthocyanins efficient. For food grade applications, ethanol is preferred over methanol. Often, organic (acetic, citric, or tartaric acids) or mineral acids (hydrochloric acid or phosphoric acid) are added to the extraction solvent to stabilize the flavylium cation. The utilization of hydrochloric acid is limited due to its potential to break down acylated anthocyanins. This restriction stems from the fact that hydrochloric acid may adversely affect the integrity of these particular compounds during the extraction process [12].

The extraction temperature plays a significant role in anthocyanin recovery, as their solubility increases at elevated temperatures. However, it is crucial to note that higher temperatures can also promote decomposition, impacting the overall efficiency of the extraction process [13]. The traditional extraction procedure consists of several extraction rounds, depending on the extractability of the system, as well as the quantities of total anthocyanins present in the matrix. For commercial applications, the extracts are then concentrated into a thick paste under a reduced vacuum in a rotavapor. Finally, the concentrated extract is freeze-dried to obtain a fine anthocyanin powder [14].

Additionally, if the extracts contain lipid materials, the addition of an organic solvent such as hexane to the extract can eliminate some lipid substances contained in said materials. Acid can cause the partial hydrolysis of the acyl moieties in acylated anthocyanins, especially those with dicarboxylic acids such as malonic acid, so the use of weak acids, such as tartaric or citric acid, is desirable to keep the dicarboxylic substituents intact [15,16]. In this sense, pH has also been shown to have a significant influence on the color of anthocyanin extracts and the absorbance readings. At lower pH values (pH < 2), blue and purple wheat extracts exhibited a red to dark red color change [12].

More sophisticated methods include those derived from the combination of sorption methods with membrane (nanofiltration, ultrafiltration, and electrodialysis) technologies [17] that provide a high extraction efficiency and allow for the selective separation of anthocyanins and other polyphenols from multicomponent natural mixtures. In recent years, technologies using ion exchange resins (IERs) or ion exchange membranes (a type of flat resin) for the extraction and purification of phenols and anthocyanins have been increasingly adopted [7].

Recently, Gravier-Rodríguez et al. [18] proposed a factorial experimental design for the ultrasound-assisted extraction of anthocyanins that allowed for a significant improvement in the amount of anthocyanins extracted. This novel procedure for anthocyanin extraction is based on the generation of cavitation bubbles, whose vibrations create fluid currents and disruptive forces in nearby cells and particles.

## 3. Biological Properties of Anthocyanins

As has been above mentioned, anthocyanins have sparked growing interest due to their bactericidal properties and a large list of unique biological benefits, such as antioxidant, anti-inflammatory, antidiabetic, chemopreventive, antimutagenic, anti-obesity, and neuro- and cardioprotective properties.

Regarding their antibacterial ability, these pigments have been noted for their ability to inhibit the growth of pathogenic bacteria, thus offering significant potential in food preservation and the prevention of foodborne illnesses. Recent research suggests that anthocyanins may act as antimicrobial agents, interfering with the cellular function of harmful bacteria and contributing to the natural preservation of food [19]. Different mechanisms have been proposed for the antimicrobial effect of anthocyanins. In this sense, it is known that anthocyanins can destroy cell wall integrity and destabilize the cytoplasmic membrane. In addition, they have also shown to negatively affect bacteria metabolism by inhibiting extracellular microbial enzymes [20].

Its outstanding antioxidant activity helps neutralize free radicals, unstable molecules that can contribute to cellular aging and various chronic diseases [21]. Indeed, anthocyanins have shown higher antioxidant properties than conventional antioxidant molecules like α-tocopherol. This important antioxidant effect derives from the special chemical structure characterized by a high number of hydroxyl groups, the catechol moiety, and the oxonium ion in the C ring. Indeed, glycosylation, which limits electrons’ delocalization, seems to decrease its free radical scavenger ability. Nevertheless, the antioxidant activity of anthocyanins can be decreased by the antagonistic interactions with other phytochemicals and vitamins also present in ingested fruits and vegetables [20,21].

Anthocyanins have been noted to possess anti-inflammatory properties, which may be especially relevant to cardiovascular health and the prevention of chronic inflammatory conditions. Inflammation is a complex biological response of injured tissue in which different external or internal stimulators, such as cytokines, interleukins, or cyclooxygenase (COX) enzymes, take part. Specifically, anthocyanins have demonstrated an inhibitory effect on COX-1 and COX-2 enzymes that is comparable with synthetic anti-inflammatory drugs such as ibuprofen and naproxen. Anthocyanins have also shown in vitro to inhibit various interleukins and NF-kβ, a nuclear factor that serves as a central inflammatory mediator. In addition, anthocyanins with an ortho-dihydroxyphenyl structure have been shown to suppress the activation of MAPK, which is related to the cellular response to proinflammatory cytokines [20].

These compounds have also been associated with improving cognitive function and a protective function against oxidative stress in the brain [18] due to their special role in inhibiting neuro-inflammation and regulating neural signaling. Different studies relate this action to a cerebral blood flow enhancement and the inhibition of NF-kβ upregulation. In vivo studies have proven that this improvement on the neuronal function results in the delay of Alzheimer’s disease, an enhancement of memory-associated neuronal signaling, and the regulation of synaptic plasticity, which is a key factor in learning and memory.

The ability of anthocyanins to modulate gene expression and regulate various metabolic pathways offers a promising outlook in the research on medical treatments and therapies. Some studies suggest that these substances may have positive effects on blood sugar regulation, which could be beneficial for individuals with diabetes (Type 2) [22].

In addition, it has been observed that anthocyanins can influence the expression of genes related to cell proliferation and apoptosis, which could have implications in the prevention and treatment of cancer [20,23]. These anticancer properties seem to be based on multiple and additive mechanisms that involve cell cycle arrest, apoptosis, anti-angiogenesis, inhibition of DNA oxidative damage, the above-mentioned inhibition of COX-2 enzymes, and anti-mutagenic and anti-carcinogenic effects. Although is widely recognized that anthocyanins play an essential role in the anticancer activity of many fruits and vegetables, several studies have shown that it is the result of the collaborative action with other phytochemicals that are also present.

In summary, anthocyanins, beyond their visual appeal in foods, have bactericidal properties that can contribute to the preservation of foods and have notable biological benefits in the human body. These properties, backed by scientific research, highlight the potential of anthocyanins in promoting health and preventing important diseases.

## 4. Halochromic Properties of Anthocyanins

Chemically, chromism is a process that induces changes, which are usually reversible, in the colors of compounds. These changes can occur due to a number of external factors, such as temperature (thermochromism), light (photochromism), electricity (electrochromism), ions (ionochromism), etc. The use of these properties in materials categorizes them as smart materials. Within the phenomenon of ionochromism, halochromism corresponds to the color change process affected by pH, being sensitive to the hydrogen ion (H^+^) (protonation or deprotonation) [24].

**Figure 3:**
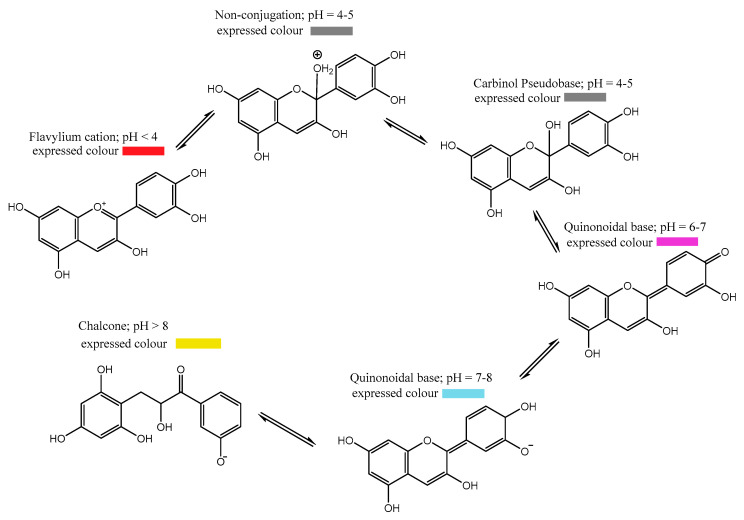
Chemical structures of anthocyanin at different pH values. Source: [25].

Anthocyanins can be found as different chemical structures and colors depending on the pH of the solution as shown in Figure 3 [25]. In general, anthocyanin displays a red color in acidic conditions, pink in neutral conditions, and blue in basic conditions [26]; thus, according to their halochromic properties, anthocyanins are excellent candidates for the development of colorimetric materials.

At pH 1, the flavylium cation (red color) is the predominant species and contributes to purple and red colors (Figure 3). At pH values between 5 and 6, only two colorless species can be observed, which are a carbinol pseudo-base and a chalcone, respectively. At pH values higher than 7, the anthocyanins are degraded depending on their substituent groups (Figure 3, degradation reaction).

At pH values between 4 and 6, four structural forms of the anthocyanins coexist: a flavylium cation, anhydrous quinoidal base, colorless carbinol base, and the pale yellow chalcone. The equilibrium between the quinoidal bases and carbinol occurs via the flavylium cation [27]. Investigations about anthocyanin stability and the color variation with pH concluded that the changes in the color of these compounds are more significant in the alkaline region due to their instability.

The ability of these substances to visually reflect variations in acidity or alkalinity makes them valuable tools in practical applications, such as the evaluation of pH in biological solutions and foods. The versatility of anthocyanins as pH indicators has increased their use in food industry because this specific capacity makes them play a crucial role in the development of non-invasive and environmentally friendly measurement methods.

## 5. Anthocyanin-Loaded Polymers: Preparation and Characterization Methods

Anthocyanin-loaded polymers have recently been a point of interest due to their potential applications as smart substrates for anthocyanin immobilization, protection, enhanced bioavailability, and controlled release. The most common matrix materials employed are biopolymers, due to their environmentally friendly, biodegradable, non-toxic, and edible nature, which makes them ideal candidates for developing nutraceutical delivery systems. In addition, naturally occurring polymers derived from renewable resources have attained attention as sustainable materials to replace petroleum-based commodity plastics traditionally used in short-term food packaging [28].

Among biopolymers, polysaccharides and proteins are the most commonly employed ones for anthocyanin loading. Both polysaccharides and proteins display excellent gelling properties that facilitate networking in hydrogel-based systems, enabling high loadings as well as remarkable film-forming properties that are essential for forming suitable films for food packaging and wound-healing applications. Polysaccharides are widely investigated and used as substrate and coating materials to encapsulate and deliver bioactive molecules. Moreover, polysaccharides show great affinity to anthocyanins, due to electrostatic, hydrogen bonding, and hydrophobic interactions, which lead to high efficiency entrapment of anthocyanins. Alginate, chitosan, gum arabic, starch, dextran, and their derivatives are the most investigated polysaccharides for anthocyanins immobilization. Proteins are also usually employed as substrate or protective materials for the encapsulation of small molecules; however, they can be easily denaturalized and have low stability, putting them at a disadvantage in comparison with polysaccharides [29]. When it comes to proteins, egg white, soy protein, gelatin, and sodium caseinate are worth highlighting due to their surface- and film-forming characteristics, while hydrophobic proteins like zein and millet protein are known to form nanoparticles by self-assembly in the appropriate conditions [29]. Plant and animal proteins show binding affinity for anthocyanins, driven mainly by Van der Waals forces, hydrophobic interactions, and covalent and H bonding.

Apart from polysaccharides and proteins, biodegradable synthetic polymers have also been explored to combine with anthocyanins. This is the case for poly (lactic acid) and polyvinyl alcohol, which have been widely proposed as biodegradable packaging films.

Since polymers can be processed into a wide variety of forms, polymer-mediated anthocyanins encapsulation has been addressed by different resourceful approaches according to the desired application. The most important encapsulation polymer systems employed in anthocyanin loading and release are described below.

### 5.1. Films

Anthocyanin-loaded polymer films are obtained mainly by solution casting, a simple and cheap technique that uses aqueous solutions or organic solvents to dissolve anthocyanins as the active agent and polymers as the substrate for immobilization [30]. In brief, polymer/s, plasticizer, and an anthocyanin extract are sequentially dissolved in solution at temperatures lower than 80 °C for a short period of time to minimize the degradation of the anthocyanins. Finally, the obtained solution is incubated onto flat Petri dishes at ~25–40 °C for 24–48 h. Other more sophisticated and expensive techniques have also been reported to fabricate anthocyanin films, such as electrospinning and layer-by-layer and 3D printing [31]. Chitosan, cellulose, starch, zein, gelatin, pectin, agarose, xanthan gum, and combinations of these have been the most commonly employed polymer systems for the development of anthocyanin-loaded films. Glycerol and sorbitol are the most popular plasticizers used in anthocyanin-based films [32].

**Figure 4:**
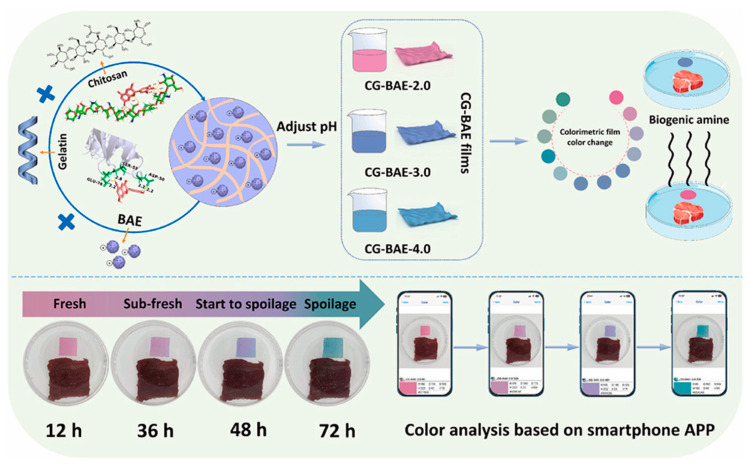
Illustrative scheme of the synthesis and performance of the smart anthocyanin-loaded chitosan/gelatin films for accurate beef sub-freshness monitoring. Reproduced with permission from Li et al. [30]. Copyright (2024), Elsevier.

Regarding films for monitoring food spoilage, it has been shown that combining polymers improves the physical properties of the material. For example, Nadi et al. [33] showed that combining basil seed gum with chitosan and adding red cabbage extract as a colorimetric indicator improves properties such as solubility, water vapor permeability, and flexibility in the material. Recently, Li et al. [30] reported the use of a film that combines chitosan and gelatin with a butterfly pea flower anthocyanin extract to evaluate beef freshness. This combination had an outstanding sensitivity to pH changes that are not perceptible to the naked eye, and the color change could be assessed using RGB values obtained from a smartphone app (Figure 4), differentiating four basic stages: freshness, sub-freshness, onset of deterioration, and complete deterioration.

Wu et al. [34] demonstrated that a novel way to promote the stability of anthocyanins in polymer films is the prior chemical modification of the phytochemical.

The incorporation of anthocyanins into films can modify the technological and functional properties of the film. These changes depend on the physical or chemical interactions between the film-forming polymer and the anthocyanin extract, which can lead to structural modifications. The interaction between biopolymers and anthocyanin-rich extracts depends on the nature, chemical characteristics, and concentration of both the polymer and extract, as well as the structural properties of the active components. The addition of anthocyanin-rich extracts can influence the thickness, color, opacity, solubility, water vapor permeability, and mechanical properties of the films [35]. Thickness is typically analyzed because, in most cases, it is modified and, since it hinders the penetration of water, it is a key parameter to take into consideration [31].

### 5.2. Mats and Fibers

It is known that nanofibrous mats have a high surface-to-volume ratio, small pore size, and high porosity compared to polymer films, which makes electrospun nanofibrous mats very attractive materials for the manufacture of substrates for the immobilization of anthocyanins [36].

The electrospinning process involves the application of an electric field to create fine polymer fibers from a solution containing anthocyanins. Anthocyanins can decrease the conductivity of the polymer solution, and consequently, as Jiang et al. [29] observed by SEM microscopy, heterogeneous fibers are formed.

Anthocyanin nanofiber mats made by electrospinning are used as tools in the field of active/smart packaging where they have been studied to improve their stability, simplify the slow and controlled release of antimicrobial and antioxidant substances [37], and as spoilage sensors [38]. When it comes to healthcare applications, nanofibrous mats of anthocyanins have found their major application as smart wound dressings that can monitor the wound healing process [39].

As an example of this, Khaledian et al. [37] reported a new material based on a potato starch–turnip peel extract and guar gum–cinnamaldehyde to control the growth of spoilage-related microorganisms by extending the shelf life of fresh lamb meat under refrigerated storage conditions for up to 13 days. More complex combinations that including fibers have been also evaluated, for example, in the case of anthocyanin nanoencapsulated in gelatin and immobilized on ethyl cellulose nanofibers reported by Nath et al. [38] that showed pH sensitivity that was stable up to 7 days of storage at room temperature.

### 5.3. Hydrogels

Hydrogels have been widely used as a method of encapsulating water-soluble active ingredients. The thermo-degradation and stability against sudden pH changes of anthocyanins could be inhibited by encapsulating them within hydrogel systems. This has been demonstrated in the process of simulated digestion and may be very useful in the prevention of intestinal diseases [40,41].

Recent research has demonstrated the versatility of these materials that allow the easy incorporation of multiple active agents. For instance, Lotfinia et al. [42] combined alginate in the form of a hydrogel with honey and red cabbage extract, and could observe improved mechanical properties, the antibacterial activity attributed to the honey, as well as the antioxidant properties and good activity against pH changes from the red cabbage.

Polysaccharides have been traditionally employed in anthocyanin hydrogel preparations due to the simplicity of their corresponding gelation process. This is the case for alginate, which forms cold gels through Ca^2+^-induced cross-linking [43], or starch [44], another widely used material for these gel-based encapsulation systems.

### 5.4. Polyelectrolyte Complexes

Polyelectrolytes are macromolecular materials that possess repeating units and dissociate into highly charged polymeric molecules in aqueous solution, forming either positively or negatively charged polymeric chains [45]. There are numerous compounds that can serve as biopolyelectrolytes such as proteins, polysaccharides, and their derivatives. Biopolyelectrolyte complexes can be formed by the titration of a biopolyelectrolyte solution in another biopolyelectrolyte solution with the opposite charge under agitation. Among the advantages of this methodology are the simplicity, quickness, and the fact that it does not require high energy, chemical crosslinkers, specialized equipment, or organic solvents. An advantage of these materials is that polymeric chains can generate dense interconnecting networks, which is useful for inhibiting the penetration of polar reactive compounds and the attack on charged anthocyanins [46].

Tan et al. [47] demonstrated that polyelectrolyte complexes obtained from chitosan and chondroitin sulfate incorporating anthocyanins and several co-pigments (catechol, gallic acid, gallic acid ethyl ester, and (-)-epigallocatechin gallate) present a synergistic effect of co-pigmentation and encapsulation in protecting anthocyanin against the nucleophilic attack of ascorbic acid. This interesting effect was attributed to the dense network created by the hydrogen bonds between the co-pigments and polysaccharides, as well as the formation of hydrophobic environments between the co-pigments and polysaccharides.

The stabilizing effect of polyelectrolyte complexes was comparatively analyzed in polycomplexes of whey protein with four different polysaccharides: chondroitin sulfate, dextran sulfate, arabic gum, and pectin. The results showed that pectin provided the highest protection, followed by arabic gum, chondroitin sulfate, and dextran sulfate. The stabilizing effects are attributed to hydrogen bonding and hydrophobic and electrostatic interactions between the whey protein and the polysaccharide [48].

### 5.5. Nanoparticles

As in the general case of polymeric nanoparticle preparations, the methods commonly used for the preparation of anthocyanin nanocarriers can be classified as the emulsification cross-linking method, ionic cross-linking method, covalent cross-linking method, and self-assembly methods.

The emulsification crosslinking method is a widely used method. A carrier particle is usually large (tens to hundreds of microns) with a highly homogeneous particle distribution. Anthocyanin nanoparticles are divided into ionically crosslinked and covalently crosslinked depending on the crosslinking method. Covalently cross-linked carriers are more stable. Studies have shown that the type of polymer employed has a relevant effect on the crosslinking efficiency. For example, pectin and carboxymethylcellulose lead to a higher crosslinking density than arabic gum with the 1-ethyl-3-(3-dimethylaminopropyl) carbodiimide/N-hydroxysuccinimide crosslinker, resulting in nanoparticles of anthocyanin with a smaller particle size (160–210 nm), more uniform distribution, high encapsulation rate (up to 80%), slow release, and much higher antioxidant activity [49].

Ionic nanoparticles could be easily formulated through complexation of two different charged biopolymers in diluted solutions. Biopolymers such as alginate, chitosan, whey, and soy protein are commonly extruded as a carrier solution through a needle or nozzle into a gelation solution containing the specified ions. This is a mild process that has shown a high encapsulation efficiency and effective protection of anthocyanins under gastric conditions [50].

### 5.6. Emulsions

Emulsion-based systems are intended to overcome the low stability and bioavailability of anthocyanins. Among the most used systems are nanoemulsions and microemulsions that have been studied in vivo and in vitro as anthocyanin delivery systems [51,52,53,54].

Nanoemulsions are colloidal dispersions formed by droplets of a liquid dispersed in another liquid that, despite being immiscible, can be stabilized with a surfactant layer. In nanoemulsions, particles showing sizes from 20 to 500 nm can avoid emulsification, flocculation, or precipitation during storage [55,56,57]. Some of the benefits of nanoemulsions are the control of the release rate and the avoidance of decomposition or degradation of the encapsulated anthocyanins [58].

Microemulsions are mainly composed of water, oil, surfactants, and cosurfactants, and have better flowability, more uniform particle dispersion, and stronger stability than emulsions. Microemulsion particles have sizes between 10 and 100 nm, which can provide stable and uniformly dispersed systems [59], which are useful properties for improving the absorption and bioavailability of anthocyanins and enable easy and multiple routes of administration [60,61].

There are several studies that report the development of nanoemulsions and microemulsions with extracts from different sources of anthocyanins, such as mangosteen peel, Brazilian berry, purple sweet potato, cranberry, red cabbage, blueberry, and jaboticaba peel, among others, showing nanoparticles that are stable for long periods of time, ranging from 30 days to 3 months [53,58,59,62,63].

**Figure 5:**
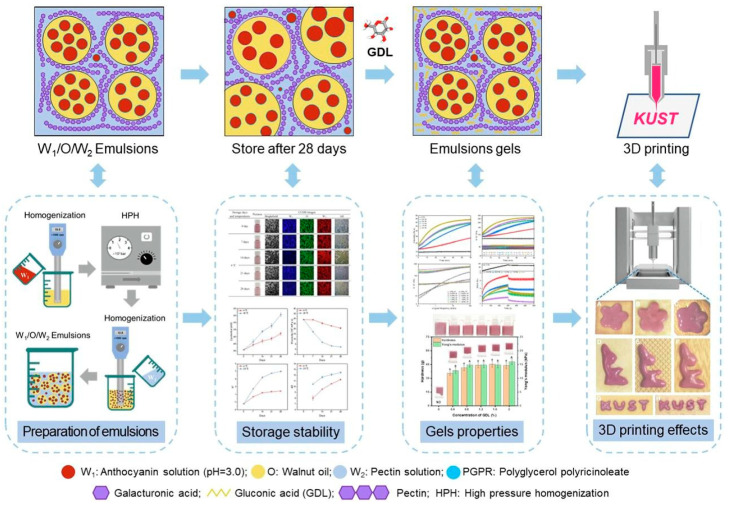
Three-dimensional printing of stable anthocyanin-loaded double emulsion gels formed by pectin and glucono-delta-lactone complexes. Reproduced with permission from Li et al. [51]. Copyright (2023), Elsevier.

Recently, many researchers have attempted to construct W1/O/W2 double emulsions designed to encapsulate anthocyanins. Among the most recent findings, it is worth highlighting a double emulsion of pectin and glucono-delta-lactone prepared by Li et al. [51] that encapsulated anthocyanins from a mulberry extract that was tested for three-dimensional printing (Figure 5). This manufacturing method has received increasing attention from the food industry due to its shape flexibility and customized design [51,64].

### 5.7. Self-Assembled Liposomes, Proteins, Peptides, and Phospholipids

Today, liposomes, phospholipids, and proteins are used as anthocyanin nanocarriers and are formed by direct self-assembly. Proteins, as was commented above, have the drawback of being very sensitive to pH changes or temperature. Liposomes also have low stability and their phospholipids are prone to oxidation during long-term storage. Phospholipids have a lot of benefits including the ability to protect sensitive ingredients, increase the bioavailability of nutrients and the efficacy of food additives, and confine undesirable flavors [65].

Amphiphilic peptides with a hydrophobic tail and a hydrophilic head are also used in molecular self-assembly. These amphiphilic peptides have good biocompatibility, biodegradable self-assembly, and chemical variability, which leads to a variety of nanostructures. Indeed, the addition of peptides to foods containing anthocyanins is recognized as the simplest way to improve the color stability of anthocyanins [58,65,66].

Recently, nanoliposomes have been reported as one of the most widely used colloidal delivery systems in food and nutrition research [58]. They are synthesized mainly from surfactants (phospholipids) that form an aqueous core and an amphiphilic lipid bilayer by embedding water-soluble substances in the aqueous phase and lipid-soluble substances in the oil phase [67,68,69]. Anthocyanins can be loaded into the internal aqueous phase of the nanoliposome to avoid degradation by light, pH, and temperature [58]. Their bilayer structure endows them with great biocompatibility, allowing them to easily fuse with bacterial membranes and penetrate microorganisms [70,71].

### 5.8. Microencapsulates

Microencapsulation consists of the incorporation of the active agent within the encapsulants by noncovalent interactions formed during a mixing process that can include additional coating layers [50]. The final drying step generally takes place by a freeze-drying or spray-drying methodology. Microencapsulation is an alternative to keep the properties of the anthocyanins intact and offer food processors a means of protecting sensitive food components [72,73,74,75,76,77]. However, during the spray drying process, some heat-sensitive anthocyanins may lose their activity or degrade and, as a consequence of the rapid dehydration, they may change their crystal structure. Consequently, freeze-drying is often preferred to mitigate these potential issues and preserve the integrity of the anthocyanins [49].

Microencapsulation allows the encapsulation of anthocyanins within a wide variety of encapsulating agents, such as polysaccharides, starches, inulin, maltodextrin or dextrose, corn syrups, arabic gum, mesquite gum, lipids, and proteins [78].

### 5.9. Specific Characterization of Anthocyanin-Loaded Polymer

The characterization of anthocyanin-loaded polymers is crucial for understanding their structure and properties. Several techniques are used to obtain significant information on these materials.

UV-Vis spectroscopy is used to analyze the absorption spectra of these materials, providing information about their specific composition, color, stability, and optimum absorption range [79].

FTIR-ATR is also a useful technique because it provides information regarding the interactions between the anthocyanin and the polymer matrix. The main changes are reported in two regions, between 1500 and 1600 cm^−1^, corresponding to the C=C bonds, and around 3200 cm^−1^, corresponding to the vibrations of the C-H bonds. In both cases, if the signals are shifted and/or widened, it confirms the immobilization of the anthocyanins in the polymer due the formation of strong interactions with the polymers [30,80,81].

Using Thermo-Gravimetric Analysis (TGA), several studies have shown that the addition of anthocyanins to polymeric matrices or the encapsulation of anthocyanins in a polymer provides greater stability with respect to pure compounds [82,83,84].

In some cases, when the main application is the detection of pH changes through color, color quantification is accomplished using the values of the L*, a*, and b* parameters. The L* value reflects the brightness; the a* value reflects the degree of red and green; and the b* values reflect the degree of yellow and blue. With the standard whiteboard as the color difference reference, the color difference value ΔE was calculated according to the following equation [85]:

(1) $$∆E=\sqrt{{\left(L-L_{0}\right)}^{2}+{\left(a-a_{0}\right)}^{2}+{\left(b-b_{0}\right)}^{2}}$$

Tassanawatm et al. [86] reported that ΔE values greater than five can be easily indicated and detected with the naked eye [87].

The most recently reported investigations about anthocyanin-loaded polymers are summarized in Table 1.

## 6. Anthocyanin-Based Polymers for Food Applications

As mentioned above, anthocyanins have been widely studied because they are bioactive substances with many attractive characteristics (antioxidant, anti-inflammatory, antibacterial, and even anticancer properties). Indeed, consuming foods high in flavonoids (of which anthocyanins are a subgroup) has multiple health benefits, such as improvements in the cardiovascular system [105], neuroprotective effects [106], and improvement in skin texture due to their antioxidant and anti-inflammatory properties [107,108]. According to this, the food industry is one of the most important areas for anthocyanin application. For this reason, recent studies have based their research on safety studies of the incorporation of anthocyanins into various polymeric matrices for their use in the food industry.

**Figure 6:**
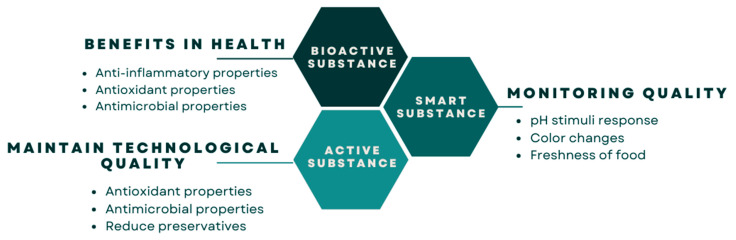
Triple role of anthocyanin-loaded polymers in food applications.

Research and food industry interest have focused around three specific targets regarding anthocyanin-loaded polymers. Firstly, improving the general health of the human body due to anthocyanins’ beneficial effects against chronic nontransmissible diseases (bioactive substance), which is reflected in the production of food products enriched with anthocyanins. Secondly, providing “intelligence” to those materials into which they are incorporated, e.g., producing so-called “smart materials” that are able to respond to external stimuli. In this sense, anthocyanins act as interactive indicators helping to assess the chemical and microbial quality of food products through pH monitoring. Finally, materials loaded with anthocyanins are “active” since they contain antimicrobial and antioxidant agents that improve food preservation and function as chemical preservatives by reducing lipid oxidation and microbial growth in packaged foods. Figure 6 shows the roles of anthocyanins as a bioactive, active, and smart substance for food applications.

### 6.1. Anthocyanins as Bioactive Substances

Bioactive substances are chemical compounds present in foods and plants that, when ingested, can benefit human health, as is the case for anthocyanins.

Due to the low bioavailability of anthocyanins, they struggle to reach the desired targets effectively. To address this issue, anthocyanin-containing edible polymers have gained significant attention. These innovative platforms withstand the upper gastrointestinal tract, resisting early molecule release or degradation due to varying pH levels and digestive enzymes. Recent studies have explored the use of different polymers for efficiently transporting anthocyanins through the human body. For example, Deng et al. [109] fabricated anthocyanin-based polysaccharide films with potential antimicrobial and antioxidant effects for food applications. They combined the wine grape pomace anthocyanin with three different plant-based polysaccharides: low methoxyl pectin, sodium alginate, and Ticafilm^®^. The controlled release of phenolic from the film matrix exhibited its potential antioxidant and antimicrobial functions. Among the synthesized films, pectin-based platforms presented the highest water solubility and a faster phenolic release rate, which make these film strong candidates to be used as an oral fast-dissolving film for pharmaceutical or nutraceutical applications.

Butkevičiūtė et al. [110] used capsules of the natural polymer gelatin to encapsulate freeze-dried apple powder as a bioactive substance to evaluate the release kinetics of phenolic compounds. Mueller et al. [111] encapsulated anthocyanins from bilberries using whey protein or citrus pectin and observed that citrus pectin encapsulation increased intestinal accessibility during passage through the small intestine [112].

### 6.2. Smart Anthocyanin-Based Polymers

Thanks to their coloring properties, anthocyanins are used as indicators to monitor and evaluate the quality of food products due to their sensitivity of their color to various factors, such as pH changes (Table 2). The detection and quantification of these substances provide valuable information about foods’ freshness, stability, and authenticity. Smart polymers are a technology, primarily used in the food industry, that incorporates materials capable of detecting immediate food spoilage. Anthocyanins have been widely used as quality indicators in packaged foods due to their sensitivity to pH changes [12], creating visual shades varying from red to purple [4].

**Figure 7:**
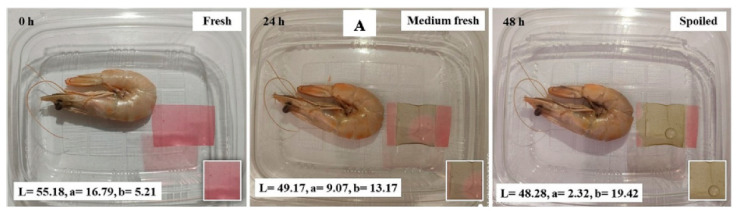
Changes in shrimp freshness during storage using intelligent halochromic films. (Reproduced with permission from Tavassoli et al. [113]). Copyright (2023), Elsevier.

Milad Tavassoli et al. [113] pioneered the utilization of novel anthocyanins sourced from sumac extracts as doping agents in pectin (PC)/chitosan nanofiber (ChNF) films. These films exhibited promising potential for monitoring shrimp decomposition by showcasing sensitivity to ammonia vapors. Notably, the intelligent film containing sumac anthocyanins showed a rapid response to volatile ammonia within the initial 5 min, as depicted in Figure 7. Furthermore, these films demonstrated remarkable antibacterial activity against *S. aureus*, *E. coli*, and *P. fluorescence* bacteria.

The same author prepared a food quality sensor by combining chitosan nanofibers and gelatin, incorporating anthocyanins from barberry fruit and saffron petals [114]. These polyphenolic compounds served as colorimetric pH-responsive indicators for monitoring fish freshness. The films notably diminished light transmission, particularly in the UV spectrum, owing to the anthocyanins’ ability to absorb UV and visible light. Both types of anthocyanins exhibited color changes corresponding to pH variations. Additionally, their study confirmed that these films effectively inhibited bacterial growth.

Similarly, Milad Bakhshizadeh et al. [115] developed pH-responsive colorimetric films as an intelligent packaging system. These films integrated gelatin along with common poppy anthocyanins (CPs) with aloe vera gel (AVG) and rosemary essential oil in order to provide the material with antioxidant and antimicrobial properties, serving as a pH sensor as well. The intelligent films exhibited color changes under different pH conditions. Another essential characteristic is that the films possessed antioxidant properties as well as antimicrobial properties against both Gram-positive and Gram-negative bacteria.

**Figure 8:**
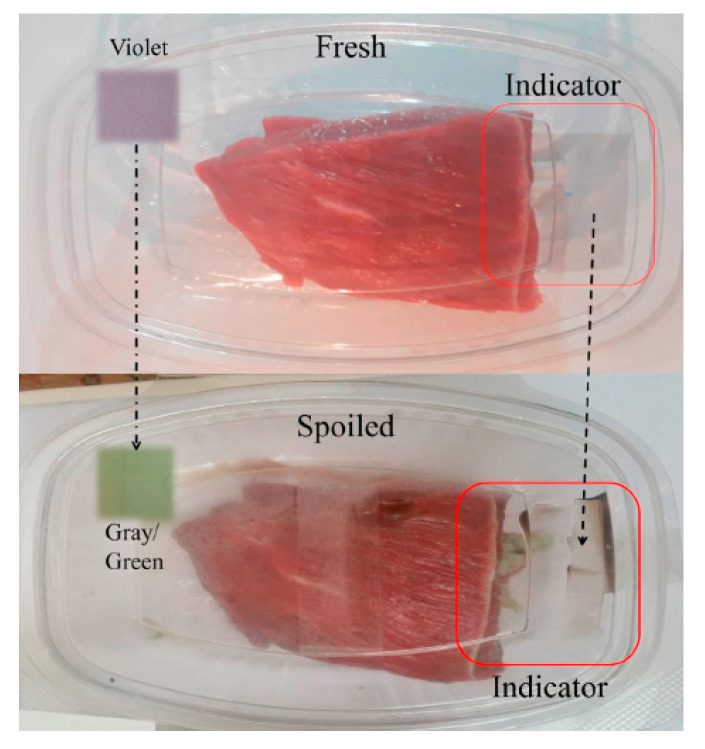
Changes in the freshness of lamb meat during storage using halochromic smart films. Reproduced with permission from Alizadeh-Sani et al. [116]. Copyright (2021), Elsevier.

Another use of anthocyanins as food quality sensors was described by Alizadeh-Sani et al. [116]. They prepared halochromic films of methylcellulose/chitosan nanofibers loaded with anthocyanins extracted from saffron petals (Figure 8). These films were mainly characterized as indicators of the quality of meat products (lamb) when used as detectors of ammonium compounds. Another essential characteristic of these materials is that they present antimicrobial properties. The antimicrobial activity of the films was stronger against *S. aureus* (Gram positive) than against *E. coli* (Gram negative).

**Figure 9:**
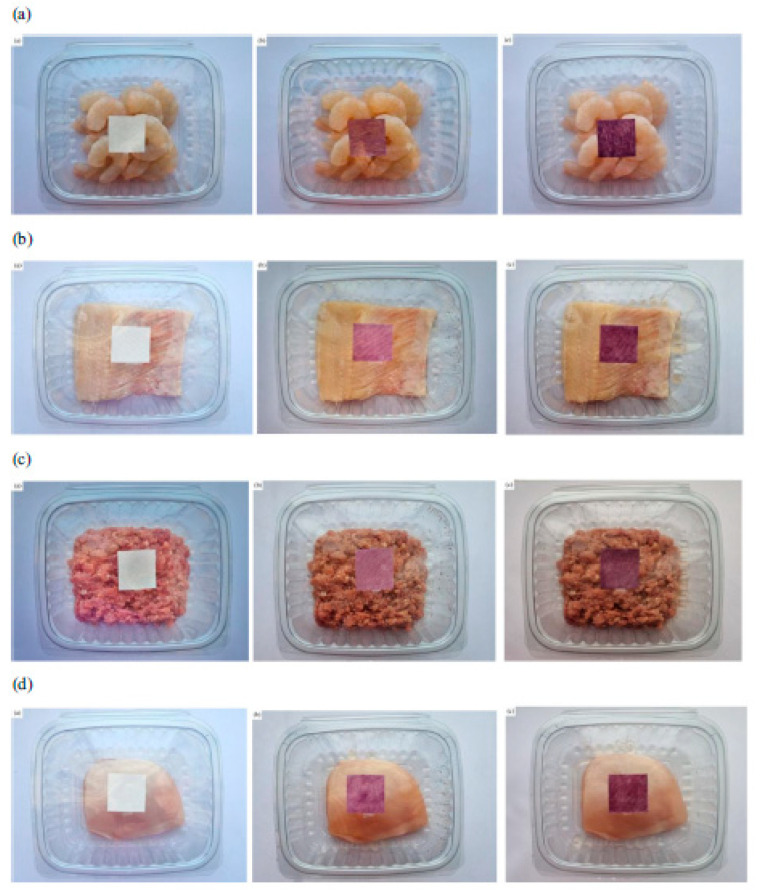
Changes in the freshness of peeled shrimps (**a**), rainbow trout fillets (**b**), minced lamb meat (**c**), and chicken fillets (**d**) under cooled conditions using CMC-PLA-VOE 5% after 0 (**a**), 3 (**b**), and 7 (**c**) days. Reproduced with permission from Fatemeh Rezaei et al. [117]. Copyright (2023), Elsevier.

Fatemeh Rezaei et al. [117] also employed anthocyanins for monitoring the freshness of different packaged foods. They synthesized double-layer polymers based on carboxymethyl-cellulose nanocrystals and polylactic acid loaded with anthocyanins extracted from *Viola odorata* petals (VOE) to control the freshness of Pacific white shrimps, minced lamb meat, chicken fillets, and rainbow trout fillets during refrigerated storage conditions. The color changes associated with VOE anthocyanins were red at pH 1–6, violet at pH 7–8, green at pH 9–10, and brown at pH 11–12; higher pH values were directly correlated with decreased freshness. Hence, these findings showed that the synthesized indicator materials could successfully monitor food freshness in real-time, as shown in Figure 9.

**Table 2:** Recent research on smart anthocyanin-based polymers in the food industry.

Source of Anthocyanins	Type of Studied Anthocyanin	Anthocyanin Role	Type of Tested Food	Polymer Matrix	Ref.
Purple corn powder	Cyanidin-3-glucoside	pH indicator for detection of NH_3_, DMA, and TMA.	Muscle food products	Alginate hydrogel beads	[43]
Roselle	Not indicated	pH indicator for real-time freshness monitoring	Penaeus vannamei (white shrimp)	PVA/HEMC/RAE/OA films	[118]
Sumac powder	Not indicated	pH indicator for detection of ammonia vapors	Shrimp	Pectin (PC)/chitosan nanofiber (ChNF) films	[113]
Powdered barberry fruit and saffron petals	Cyanidin-3-glucoside	pH indicator for detection of ammonia vapors	Fish	Gelatin/chitosan nanofibers films	[114]
Common poppy	Not indicated	pH indicator for detection of ammonia vapors.	Fish	Gelatin (G)/rosemary essential oil (REO)	[115]
Saffron petals	Cyanidin-3-glucoside	pH indicator for detection of ammonia vapors	Meat product (lamb)	Methyl cellulose/chitosan nanofibers film	[116]
*Viola odorata* petals	Delphinidin-3-(4-p-coumaroyl)- rutinoside-5-glucoside and cyanidin-3-O-glucoside	pH indicator for detection of ammonia vapors	Pacific white shrimps, minced lamb meat, chicken fillets, and rainbow trout fillets	Double-layer polymers based on carboxymethyl cellulose/cellulose nanocrystals and poly(lactic acid)	[117]
*Lycium ruthenicum* anthocyanins	Not indicated	pH indicator for detection of volatile acids	Milk	Alginate-konjac/glucomannan films	[119]
Red cabbage (*Brassica oleracea* var. capitata f. rubra)	Not indicated	pH indicator for detection of ammonia vapors	Not studied	Chitosan/chitin nanocrystals with curcuma oil	[120]
*Jacaranda cuspidifolia* petals	Not indicated	pH indicator for detection of ammonia vapors	Fish	Chitosan/polyvinyl alcohol	[121]

### 6.3. Active Anthocyanin-Based Polymers

Anthocyanins have sparked interest in food preservation due to their antioxidant and antimicrobial properties [122] and ability to slow the oxidation and deterioration of certain products. Firstly, due to their antioxidant activity, they act as free radical scavengers, helping prevent lipids and protein oxidation in foods [21]. This is particularly relevant to avoid the rancidity of oils and maintain the quality of high-fat products [123]. Secondly, they act as oxidation inhibitors, a process that can cause changes in the flavor, color, and texture of foods [124]. The ability of anthocyanins to inhibit oxidation makes them helpful agents for preserving the freshness and quality of products such as oils, meats, and dairy products [125]. They also have antimicrobial properties, which means they can help inhibit the growth of microorganisms, such as bacteria and fungi, in food [126]. This can contribute to a longer shelf life and microbiological safety of products. Another important factor is color preservation, as products [12] may lose color due to exposure to air, light, or temperature changes. For this reason, various authors have focused their research on incorporating anthocyanins into different polymer matrices for active packaging. For instance, Rashid et al. [127] developed an innovative antibacterial food colorant leveraging anthocyanins through microencapsulation to enhance food preservation. They utilized anthocyanins extracted from *Clitoria ternatea* and incorporated them onto maltodextrin polysaccharide, an extensively employed food additive known for enhancing flavors. In vitro bactericidal assays concluded that the microencapsulated anthocyanins can inhibit the growth of different foodborne Gram-negative bacteria, showcasing the potential of these complexes as promising antibacterial food colorants for food safety and preservation applications.

Similarly, Wagh et al. [128] fabricated an antibacterial and UV-blocking film tailored for active and smart food packaging applications, which employed anthocyanins and carbon dots (CDs). To produce these films, several anthocyanins from *Brassica oleracea* were extracted and combined with cellulose nanofibers and CDs. The results confirmed that the addition of anthocyanins significantly enhanced the antioxidant properties compared to those films based only on cellulose. Moreover, in vivo studies demonstrated the real-time monitoring capability of these films and their ability to track the freshness of different foods like pork, fish, or shrimps. These findings underscore the potential of these active films in extending food freshness, while also serving as a suitable tool for monitoring food quality.

Again, anthocyanins and CDs were utilized in food packaging by Khan et al. [122]. They synthesized a carrageenan-based multifunctional film incorporating anthocyanins and Zn-doped CDs to monitor the freshness of shrimps and enhance the utility of the films as a packaging material. According to the antioxidant and antimicrobial tests, both Zn-doped CDs and anthocyanins notably improved the antioxidant properties of the films. Meanwhile, the antibacterial effect was particularly prominent upon the addition of Zn-CDs. Additionally, the incorporation of anthocyanins provided color variations based on pH changes, directly indicating the freshness of the food. These finding suggest that these films possess the capacity to extend shrimp preservation by reducing oxidation and microbial growth while actively monitoring material spoilage. Hence, these films have emerged as an excellent alternative for dual-purpose applications, serving as both smart and active packaging solutions.

More examples of the use of anthocyanins for food preservation and spoilage reduction are described in Table 3.

## 7. Anthocyanin-Based Polymers for Healthcare Applications

Owing to the vibrant and diverse color spectrum of anthocyanins, they have found multiple applications as natural dyes in various healthcare-related fields. These natural compounds present an intriguing potential as safe and biocompatible alternatives to synthetic dyes. Due to their ability to exhibit color shifts under different conditions, their potential use as biosensors has garnered attention in recent years. In addition, anthocyanins show excellent antioxidant and anti-inflammatory effects as well as potential in the prevention of cancer, neurodegenerative diseases, and diabetes, making them very interesting natural bioactive compounds. However, their limited bioavailability remains as one of the main disadvantages for this family of compounds. Therefore, the development of systems to increase the bioavailability and, consequently, the efficacy of anthocyanins is desirable. This section will focus on two key aspects: the utilization of anthocyanins as biosensors in biomedical applications (Table 4) and the prevalent nanoencapsulation approaches designed to enhance their efficacy through controlled delivery systems (Table 5).

### 7.1. Biosensors

**Figure 10:**
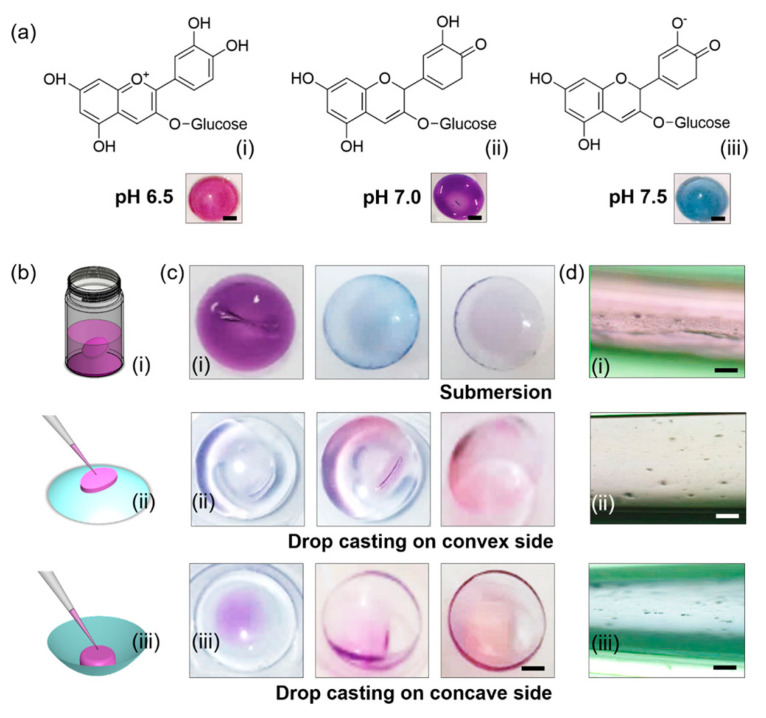
Preparation of the anthocyanin-loaded pH-sensing soft contact. (**a**) Anthocyanin chemical formulas at physiological pH levels: (i) pH 6.5, (ii) pH 7.0, and (iii) pH 7.5. Scale bars: 5.0 mm. (**b**) Illustration of the different preparation methods: (i) soaking and (ii,iii) drop-casting on the convex/concave face of the contact lenses. (**c**) Color changes in contact lenses according to the preparation method. Scale bars: 5.0 mm. (**d**) Micrographs of the cross sections of loaded contact lenses at the different pHs: (i) pH 6.5, (ii) pH 7.0, and (iii) pH 7.5. Scale bars: 200 μm. Reproduced with permission from Riaz et al. [131]. Copyright (2019) CC, ACS Publications.

Anthocyanins exhibit changes in their chemical structure in response to varying hydrogen ion concentrations. This characteristic has garnered extensive attention for utilizing anthocyanin pigments as key components in biocompatible pH sensors. Riaz et al. [131] studied the use of anthocyanins on contact lenses to monitor the ocular pH, a crucial parameter in evaluating ocular health post-eye surgery in conditions like keratoconjunctivitis and ocular rosacea. For this purpose, commercially available lacreon and lacreon-free contact lenses were functionalized with the anthocyanins obtained from *Brassica oleracea* by soaking and drop casting methods. The lacreon contact lenses demonstrated enhanced and uniformly distributed coloration that was noticeable to the naked eye within the physiological pH range of 6.5 and 7.5 (Figure 10).

Similarly, Alsahag et al. [132] developed an economical, reversible, eco-friendly wound dressing based on anthocyanins in order to monitor wound healing progress. This innovative bioassay detected pH changes in a simulated wound solution, which were indicated by shifts in color. They employed anthocyanins sourced from *Brassica oleracea* and L. var capitate that were integrated onto a carboxymethyl cellulose/polyvinyl alcohol composite. The comfort and durability of these composites were confirmed through favorable colorfastness, air permeability, and bend length. In terms of biological properties, these composites exhibited non-cytotoxic effects and enhanced antimicrobial properties. This colorimetric assay offers an affordable, used-friendly sensor for monitoring wound healing progress. In contrast to prior electric-based sensing tools requiring complex equipment, this chromogenic sensor enables onsite wound pH measurements without intricate procedures.

Another example of anthocyanin-based materials used as biosensors was reported by Al-Qahtani et al. [133]. They encapsulated both natural anthocyanin and urease enzyme in a calcium alginate biopolymer, which were then immobilized within the fabric. The resulting bio-chromic sensor offered quick responses with a detection limit of 300–1000 ppm for urea. This innovative reversible sensor employs urease to convert urea to ammonia, allowing for easy urea detection via the encapsulated anthocyanin pH indicator from red cabbage. This method provides an efficient, eco-friendly, and selective colorimetric approach for measuring urea levels in fluids like blood and urine, demonstrating a potential for practical biomedical applications.

The same authors described the development of a sponge-like colorimetric swab by immobilizing red cabbage extract anthocyanins onto a cellulose aerogel [134]. This biochromic device was synthesized in order to monitor sweat. Sweat is a fluid emitted by the skin and can provide valuable insight into an individual physiology. For instance, in drug testing, sweat might contain measurable amounts of opioids like cocaine, gamma hydroxybutyrates, amphetamines, buprenorphine, and cannabinoids, which can alter pH levels. This makes sweat a significant factor in forensic science and toxicology. When the sponge-like device was exposed to a sweat solution, its color shifted from pink (620 nm) to greenish yellow (529 nm), corresponding to the pH of the solution. The aerogel biosensor displayed remarkable sensitivity and it can provide a real-time sweat analysis. Additionally, they observed that a sponge rich in anthocyanin exhibited no harmful effects on human skin. This eco-friendly biosensor offers a simple, reversible way to detect perspiration conditions using a sponge made from natural cellulose and anthocyanin, making this device great for drug testing applications.

### 7.2. Nanoencapsulated Delivery Systems

Taking into account the inherent toxicity associated with numerous synthetic drug compounds, the discovery and development of novel and efficient bioactive natural compounds like anthocyanins is highly desirable. However, since these natural phytochemicals are remarkably sensitive to external environmental factors, resulting in a notably brief half-life, as has been described above, the development of innovative delivery systems that can provide stability to anthocyanins without compromising their bioactivity is crucial. Indeed, it is essential to achieve kinetic and thermodynamic stability while simultaneously enhancing solubility and improving bioavailability [135].

Among the diverse array of alternatives explored, encapsulation has garnered substantial attention in recent decades, showcasing immense potential in various sectors, including the pharmaceutical and nutraceutical industries [136]. Furthermore, nanoencapsulation strategies exhibit interactions, such as Van der Waals forces, hydrophobic interactions, and hydrogen bonding, between nanocarriers and natural compounds like anthocyanins, ensuring enhanced material stability and increased bioavailability. Indeed, these advantages endow nanomaterials with the remarkable ability to easily traverse the blood–brain barrier, thereby magnifying the therapeutic potential of the encapsulated molecules [135].

This section highlights the application of three different polymer types, polysaccharides, lipids, and synthetic polymers, for nanoscale coatings of anthocyanin. Polymeric materials used in nanoencapsulation can be classified into two main categories based on their sources: bio-based polymers, from plant or animal sources, and oil (petroleum)-based polymers, from petroleum feedstock. Owing to the defined chemical structure, synthetic polymers offer control over their physical and chemical properties compared to natural polymers. Conversely, bio-based polymeric nanoencapsulations offer key advantages for use in biomedical applications as they present remarkable biocompatibility across a wide range of concentrations and are often cost-effective [137]. In nanomedicine, these polymers predominantly fall into two subcategories: polysaccharides and proteins. It is important to highlight that certain polysaccharides are commonly used alongside anthocyanins due to their inherent charged form. This ionization enables effective interaction with oppositely charged phospholipids, effectively preventing phospholipid hydrolysis under acidic pH conditions or in the presence of enzymes. Chitosan and pectin stand out among these polysaccharides as the most commonly used due to their non-toxic, eco-friendly, biodegradable nature and their high biocompatibility. Apart from these inherent advantages, they demonstrate considerable potential as an efficient drug delivery system, particularly for targeting the colon. For instance, Zhao et al. [138] studied the activity of anthocyanins encapsulated in pectin and chitosan and the controlled release from these nanoparticles, demonstrating enhanced protection for normal rat kidney cells against acrylamide-induced damage. Additionally, they decrease reactive oxygen species as well as matrix metalloproteinases and glutathione levels, providing protection to normal human hepatocyte L02 cells against palmitic acid-induced damage. Nevertheless, it has to be noted that chitosan dissolves only in certain dilute acidic conditions.

Similarly, Sreerekha et al. [139] also employed chitosan to encapsulate anthocyanins and demonstrated excellent antioxidant effects in vitro. They further studied the hypolipidemic effect and the potential dietary supplementation of these nanoparticles in male Wistar rats. The study revealed that these nanoparticles effectively reduced serum total cholesterol and triglyceride levels. Moreover, they mitigated lipid-mediated stress and lowered lipid levels in both the serum and liver. Thus, this highlights the potential of anthocyanin-loaded nanoparticles as functional dietary supplements with definite hypolipidemic effects.

In the same way, Amararathna et al. [140] proposed the use of different polymers to enhance the effectiveness of anthocyanins, by developing a direct delivery method to lung tissues. They employed anthocyanins from ripe haskap berries (HB) and encapsulated them in three types of nanocarriers: polyethylene glycol-poly(lactide-co-glycolide), maltodextrin, and carboxymethyl chitosan (CMC). Among these three nanocarriers, CMC showed the highest encapsulation efficiency at 10%. They tested the cytotoxicity and protective effects of the CMC-encapsulated anthocyanins on lung cells and found no harmful effects. Additionally, HB anthocyanins loaded into CMC helped counteract the oxidative stress caused by carcinogens, restoring the activity of important enzymes. Moreover, in in vivo tests with mice with inhalation exposure to HB-CMC for six days showed the presence of anthocyanins in the lungs within an hour. This suggests that CMC could be a safe encapsulating agent, facilitating the direct delivery to lung tissues and potentially improving therapeutic effectiveness by slowing down metabolic processes.

Another promising polysaccharide explored for the encapsulation of anthocyanins is chondroitin sulfate. Jeong et al. [141] reported the synthesis of black soybean anthocyanins loaded at different concentrations onto chondroitin sulfate polysaccharides in order to improve the structural stability of this natural antioxidant. When compared to anthocyanins alone, these nanoparticle complexes demonstrated superior inhibition in human cervical cancer HeLa cells. Additionally, Liang et al. [142] demonstrated that the combination of chondroitin sulfate and chitosan with loaded black rice anthocyanins induced apoptosis of human HCT-116 colon cancer cells by providing negative charges to the mitochondria. The results show that the addition of anthocyanin exhibited a noteworthy reduction in cell viability, which altered the mitochondrial structure and, consequently, increased apoptosis of cells.

Hyaluronic acid (HA) stands out as another promising natural polysaccharide that has demonstrated enhanced bioavailability and efficacy in encapsulating anthocyanins. In a study conducted by Liu et al. [143], a black rice anthocyanin-loaded HA nanocarrier was developed to reduce xanthine oxidase (XO) activity, a crucial enzyme involved in the generation of reactive oxygen species and the production of uric acid. In a simulated in vitro analysis, the anthocyanin-embedded HA nanocomplex exhibited a rapid release within the first 12 h, which was maintained consistently until reaching 60% release after 60 h, marking a 54 h difference compared to the non-embedded anthocyanin release system. These results indicate that sustained release could reduce the degradation of active compounds, thereby potentially improving the bioavailability of anthocyanins. Furthermore, in vitro assays concluded that the synthesized nanocomposite has potential to inhibit XO activity, suggesting a potential reduction in uric acid levels.

Another polysaccharide example was described by Hanafy et al. [96]. They developed a nanoparticle hydrogel platform based on starch corn, a natural polymer that has garnered attention in recent years. The anthocyanin-loaded starch hydrogel demonstrated promising therapeutic potential in eliminating glycogen from cardiac tissues, overcoming cardiomyopathy, and reducing malondialdehyde levels and collagen fibers. These findings highlights the significance of using biodegradable nanocarriers for encapsulating anthocyanins, showcasing their potential application across various biomedical fields.

On the other hand, much like other drug and active compounds, synthetic polymers have been found to be an excellent approach for the encapsulation of anthocyanins. These polymers offer versatility and enable precise control over the mechanical properties of the material. Among the synthetic polymers, polyesters have been extensively employed in anthocyanin delivery systems. For instance, biodegradable poly(lactic-co-glycolic acid) acid (PLGA) has been used to coat anthocyanins such as pelagonidin, resulting in improved protection against mitochondrial dysfunction [144]. Moreover, it has been demonstrated that the nanoencapsulation of pelargonidin anthocyanin by PLGA can inhibit the production of reactive oxygen species.

Similarly, Amin et al. [145] encapsulated anthocyanins within biodegradable nanoparticles formulated with PLGA and a stabilizing agent, polyethylene glycol (PEG-2000). They concluded that this novel approach bolsters the free radical scavenging abilities of anthocyanins and increases cellular uptake and bioactivity in in vitro assays, even at high doses, without inducing cytotoxicity. They investigated the biological activity and neuroprotective effects of these anthocyanin-loaded nanoparticles (An-NPs) in SH-SY5Y cell lines. The results indicated that anthocyanin-loaded nanoparticle systems effectively mitigated AB-induced neurotoxicity in SH-SY5Y cells, displaying notable antioxidant, anti-apoptotic, and anti-inflammatory properties and prevented Alzheimer’s disease. Overall, these findings suggest that anthocyanin-loaded nanoparticle systems can be a potential approach to treat various neurological disorders, including Alzheimer’s disease.

Regarding the use of other polyesters, in this case polyethylene oxide (PEO), Giaconia et al. [146] developed an anthocyanin-loaded nanofiber composite in order to enhance the antioxidant effect. Anthocyanin compounds obtained from jussara pulp were combined with polyethylene oxide (PEO) and deposited by electrospinning. Subsequently, they were subjected to a simulated digestion process in order to observe the protective effect of these natural compounds. These nanofibers demonstrated superior protection for the bioactive compounds, ensuring increased antioxidant activity compared to lyophilized jussar pulp fibers. Overall, the use of electrospinning to form fibers revealed that the composites could maintain their antioxidant effect even after digestion making these polymeric substrates promising materials to use in food so as to preserve the biological effect of jussara anthocyanins.

On the other hand, Ghiman et al. [147] described the use of a non-biodegradable polyester, polyacrylic acid (PAA), to develop fluorescent nanocarriers loaded with anthocyanins within melanoma cells. For this purpose, chokeberries anthocyanins were encapsulated between PAA layers, generating a polyelectrolyte system between PAA and poly(allylamine hydrochloride)(PAH), where rhodamine B, a fluorophore, was firstly added. According to the in vitro test, the anthocyanin fluorescent system demonstrated that could be used for trafficking over 24 h without compromising the proliferation of melanoma B16-F10 cells. The presented data not only demonstrated the potential of anthocyanin encapsulation, but also offer valuable insight into an innovative formulation for delivering therapeutic molecules or bio-imaging agents directly to melanoma cells.

**Figure 11:**
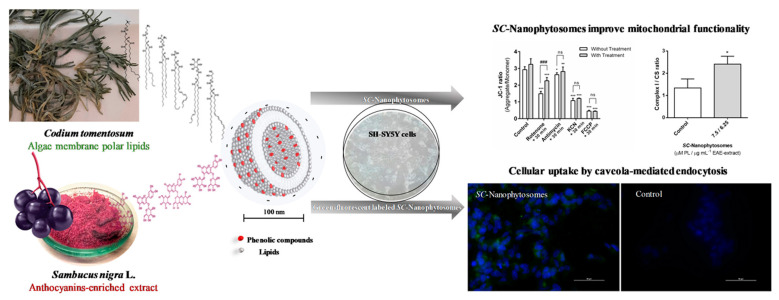
Nanoencapsulates of anthocyanin-enriched extract formed by lipid assembly generated by Mendes et al. [148] showed successful cellular uptake and improved mitochondrial functionality. Reproduced with the permission of Mendes et al. [148], licensed under CC BY-NC-ND 4.0 DEED. Copyright (2021), Elsevier.

Regarding lipid-based nanoparticles, several studies of anthocyanin nanoencapsulations have been reported. For instance, Mendes et al. [148] studied the nanoencapsulation of anthocyanins derived from elderberries (*Sambucus nigra*) by combining them with membrane polar lipids through self-assembly. These nanophythosomes presented a remarkable ability to enhance complexes I and II required in the mitochondrial respiratory chain. Additionally, they preserved the mitochondrial membrane in the presence of rotenone, by protecting the cells against rotenone- and glutamate-induced toxicity. Overall, the results suggest that these nanocomplexes are an interesting approach for facilitating targeted mitochondrial therapy in the treatment of neurodegenerative diseases (Figure 11).

Similarly, Fidan et al. [149] reported the attachment of anthocyanins from black carrots onto niosomes, lamellar structures made by non-ionic surfactants from the alkyl polyglycerol ether class, in order to improve the bioavailability of these natural bioactive compounds. The in vitro results suggested that the majority of the encapsulated anthocyanin was released after 10 h, decreasing the viability of Neuro 2A neuroblastoma cells. Overall, these findings indicate that niosomes can serves as promising vesicles for anthocyanin delivery systems. On the other hand, Priprem et al. [150] discovered that anthocyanins extracted from *Zea mays* and *Clitoria ternatea* stimulated higher collagen production in human gingival fibroblasts, which can transfer through the esophageal mucosa. In this study, anthocyanins were subjected to nanoclusters, forming crystalline-like aggregates through both intra- and inter-molecular interactions. These nanocomplexes were encapsulated, again, in niosomes and were then integrated into a mucoadhesive gel. According to the in vitro assays, the permeability of the anthocyanins was improved and enhanced due to noisome encapsulation. Moreover, the anthocyanin-loaded noisome gel demonstrated an anti-inflammatory effect and induced oral wound closure in rats. This effect could be attributed to enhanced mucosal permeability and the presence of the anthocyanins released from the noisome gel.

## 8. Conclusions and Future Outlook

Anthocyanins have significantly impacted human health and the food industry due to their halochromic and valuable biological properties. However, their low bioavailability and unstable nature are large handicaps during the storage, processing, and uptake stages, which restrict their extensive applications and require additional strategies for protecting anthocyanins. Interestingly, the incorporation of anthocyanins into polymer matrices has resulted in an effective method not only to immobilize and protect these molecules, but also to promote their controlled release. Fortunately, there are many and varied general methods of processing polymers to facilitate the encapsulation of anthocyanins. These polymer-based combinations offer many possibilities to create materials with tailored physicochemical properties, which is especially interesting in addressing anthocyanin-related concerns in the food and healthcare sectors.

This review outlines the main polymer-based encapsulation systems designed to enhance the stability and bioavailability of anthocyanins. These systems are based on diverse processing forms of polymers, such as films, emulsions, hydrogels, fibers, microcapsules, nanoparticles, self-assembled structures, and polycomplexes.

Regardless of their processed form, the recent research on anthocyanin-loaded polymer systems has mainly focused on their immobilization and subsequent controlled release, and on their application as colorimetric sensors. The food industry, in particular, has focused on developing anthocyanin polymer films for smart food packaging. These films have shown an effective performance as food quality indicators, antioxidant and antimicrobial packaging to protect against microorganisms, and active films promoting health benefits for consumers.

With respect to the healthcare field, the color-sensing property of anthocyanin-loaded polymers has been exploited to develop chromic biosensors to monitor tissue healing processes, detect the presence of sweat or urea, and prevent chronic diseases. In addition, anthocyanin-loaded nanoparticles have also been intensively investigated as a new, effective, and safe administration route for anthocyanins in preventing chronic diseases.

Despite the extensive literature on anthocyanin-based polymer systems, there are still huge challenges such as improving the processing methods to effectively protect these molecules and increase their bioavailability. These challenges offer plenty of research opportunities that encompass different approaches such as improving the efficiency of the encapsulation, reducing the amount of reagents used, and promoting the sustainable release of anthocyanins, their local delivery at the absorption sites, or their responsive release under specific triggers.

On the other hand, the biodegradability of the employed polymer/s, and the sustainability and economic viability of the processing of anthocyanin-loaded polymers are still challenging issues for future industrial applications. Exploiting current biodegradable alternatives and processing methods has resulted in low yields and is not economically feasible for the food and healthcare industries. Thus, interdisciplinary efforts to explore the opportunities for the conservation of anthocyanin properties, together with the development of environmentally friendly polymer matrices and cost-effective and scalable processes, are required to achieve the successful and widespread application of anthocyanin-loaded polymeric systems in the food and healthcare fields.

## Figures and Tables

**Table 1:** Some recently reported anthocyanin-loaded polymer systems.

Type of Material	Anthocyanin Function	Extract Used	Ref(s).
Aerogel	pH indicator	Red grape	[9]
Cryogel	pH indicator	Red radish	[88]
Double emulsions	Bioactive substance	Mulberry	[9]
Double-layer nanofiber mats	pH indicator	Turnip peel extract and potato starch–turnip peel extract	[37]
Electrospun fiber mats	pH indicator	Prunus domestica	[89]
Electrospun film	pH indicator	Blueberry	[90]
Nanoencapsulation/spray drying	Active ingredient	Aronia	[85,91]
Film	pH indicator	Black carrot	[92,93]
Film	pH indicator	Red cabbage	[94,95]
Hydrogel	pH indicator	Red beets, purple corn	[43,96]
Hydrogel	CO_2_-sensitive indicator	Black goji anthocyanin	[97]
Hydrogel	pH indicator	Aronia, liriope, and red cabbage	[98]
Hydrogel	Bioactive substance	Blueberry	[40,83]
Film	pH indicator	Red cabbage	[87]
Lipid nanoparticles	Bioactive substance	Purple sweet potato	[99]
Microencapsulation spray drying	Bioactive substance	Taro tubers	[100]
Microencapsulation spray drying	Bioactive substance	Chokeberry	[73]
Nanocoating	Colorimetric sensor	Cyanidin 3-O-glucoside	[100]
Films	pH indicator	Black rice	[101]
Nanoliposomes	Bioactive substance	Grape skin	[66]
Emulsion	Bioactive substance	Black rice	[102]
Polyelectrolyte complex	pH indicator	Non-pomace residue of grape juice powder	[103]
Polysaccharide assembly	Bioactive substance	Purple corn cob	[104]

**Table 3:** Recent research on active anthocyanin-based polymers in the food industry.

Source of Anthocyanins	Polymer Matrix	Effects/Results	Reference
*Clitoria ternatea* flowers	Maltodextrin	Enhanced stability. Improved antibacterial effect.	[127]
*Brassica oleracea* (BO) extract	Cellulose nanofiber loaded with carbon dots	Reduced spoilage. Enhanced antioxidant effect. Improved thermal stability. UV barrier properties.	[128]
Pomelo peel	Fish scale gelatin (FSG)/alginate dialdehyde (ADA) loaded with carbon dots	Physiological qualities post-harvest and extended shelf-life.	[129]
Red cabbage (*Brassica oleracea*)	Gelatin/poly(vinyl alcohol)-based matrix integrated with metal–organic frameworks	Improved antibacterial and antioxidant effects. Spoilage detection. Enhanced shrimp preservation.	[130]
Purple kohlrabi peel	Carrageenan-based matrix containing Zn-carbon dots	Improved antioxidant and antimicrobial properties. Reduced spoilage. Excellent sensor for food quality.	[122]

**Table 4:** Recent applications of anthocyanins as biosensors in the biomedical field.

Source of Anthocyanins	Type of Anthocyanin	Anthocyanin Role	Polymer Matrix	Applications	Ref. *
*Brassica oleracea*	Not indicated	pH indicator	ACUVUE^®^ contact lenses and poly(hydroxyethyacrylamide)	Ocular biosensor	Riaz et al. [131]
*Brassica oleracea* L. Var. capitata	Not indicated	pH indicator	Carboxymethyl cellulose (CMC) and polyvinyl alcohol (PVA)	Wound dressing	Alsahag et al. [132]
Red cabbage	Not indicated	pH indicator	Calcium alginate and cellulose	Urea detection	Al-Qahtani et al. [133]
Red cabbage	Not indicated	pH indicator	Cellulose	Sweat fluid detection	Al-Qahtani et al. [134]

Ref. *: references.

**Table 5:** Anthocyanin-loaded nanoparticle activity and applications in different healthcare areas.

Anthocyanin Source	Anthocyanin Type	Polymer Matrix	Advantages	Application(s)	Ref.
Bilberry	Not indicated	Chitosan and pectin	Improved bioavailability Gastrointestinal protection	Nutraceutical	Zhao et al. [138]
Black carrot	Not indicated	Chitosan	Induced hypolipidemic effect	Nutraceutical as dietary supplement	Sreerekha et al. [139]
Haskap berry (*Lonicera caerulea* L.)	Cyanidin 3-O-glucoside (C3G)	PLGA, maltodextrin, and CMC	Reduced carcinogen-induced oxidative stress	Anticancer therapy	Amararathna et al. [140]
Black soybean	Not indicated	Chondroitin sulfate	Inhibited proliferation of cancer cells (HeLa)	Anticancer therapy	Jeong et al. [141]
Black rice	Not indicated	Chitosan/chondroitin sulfate	Improved gastrointestinal bioavailability Reduced cancer cell viability Induced colon cancer cell apoptosis	Nutraceutical, functional foods, and anticancer therapy	Liang et al. [142]
Black rice	Cyanidin-3-glucoside)	HA	Enhanced stability Reduced xanthine oxidase activity	Nutraceutical	Liu et al. [143]
Not indicated	Not indicated	Starch from corn	Reduced glycogen levels Improved cardiomyopathy	Cardiovascular diseases	Hanafy et al. [96]
Not indicated	Pelargonidin	PLGA	Increased protection and control of mitochondrial dysfunction	Nutraceutical for diabetes prevention	Samadder et al. [151]
Not indicated	Not indicated	PLGA/PEG-2000	Protection against free radicals Excellent antioxidant, antiapoptotic, and anti-inflammatory effects Protection against Alzheimer’s disease	Prevention and treatment of neurological disorders	Amin et al. [145]
Jussara pulp	Not indicated	PEO	Improved thermal stability and enhanced antioxidant effect	Nutraceutical and food preservation	Giaconia et al. [146]
Chokeberries	Not indicated	PAA and PAH	Improved anti-tumoral performance Monitor and trafficking	Anticancer therapy and biosensor	Ghiman et al. [147]
Elderberries (*Sambucus nigra*)	Not indicated	Lipids (from *Codium tomentosun*)	Improved protection of mitochondrial membrane	Prevention and treatment of neurodegenerative diseases	Mendes et al. [148]
Black carrots	Not indicated	Niosome (cholesterol and non-ionic surfactants)	Improved bioavailability Reduced neuroblastoma cell viability	Pharmaceutical and biotechnological applications Anticancer therapy	Fidan et al. [149]
Black rice (*Zea mays* and *Clitoria ternatea*)	Not indicated	Niosome (cholesterol)	Enhanced bioavailability Promoted collagen production Improved anti-inflammatory effect	Wound healing systems	Priprem et al. [150]

## Data Availability

Not applicable.
